# Complexities in Defining the Unit of Intervention for Reactive Community-Based Malaria Treatment in the Gambia

**DOI:** 10.3389/fpubh.2021.601152

**Published:** 2021-02-26

**Authors:** Fatou Jaiteh, Joan Muela Ribera, Yoriko Masunaga, Joseph Okebe, Umberto D'Alessandro, Julie Balen, Jane Achan, Rene Gerrets, Koen Peeters Grietens

**Affiliations:** ^1^Medical Research Council Unit the Gambia at the London School of Hygiene and Tropical Medicine, Banjul, Gambia; ^2^Medical Anthropology Unit, Institute of Tropical Medicine, Antwerp, Belgium; ^3^Faculty of Social and Behavioural Sciences, Amsterdam Institute of Social Science Research, Amsterdam, Netherlands; ^4^PASS Suisse, Neuchâtel, Switzerland; ^5^Liverpool School of Tropical Medicine, Liverpool, United Kingdom; ^6^London School of Hygiene and Tropical Medicine, London, United Kingdom; ^7^School of Health and Related Research (ScHARR), The University of Sheffield, Sheffield, United Kingdom; ^8^School of Tropical Medicine and Global Health, Nagasaki University, Nagasaki, Japan

**Keywords:** malaria elimination, asymptomatic infections, reactive intervention unit, household conceptualization, transdisciplinary research

## Abstract

With significant declines in malaria, infections are increasingly clustered in households, or groups of households where malaria transmission is higher than in surrounding household/villages. To decrease transmission in such cases, reactive interventions target household members of clinical malaria cases, with the intervention unit (e.g., the “household/s”) derived from an epidemiological and operational perspective. A lack of unanimity regarding the spatial range of the intervention unit calls for greater importance to be placed on social context in conceptualizing the appropriate unit. A novel malaria elimination strategy based on reactive treatment was recently evaluated by a cluster randomized trial in a low transmission setting in The Gambia. Transdisciplinary research was used to assess and improve the effectiveness of the intervention which consisted, among others, of reflecting on whether the household was the most adequate unit of analysis. The intervention was piloted on the smallest treatment unit possible and was further adapted following a better understanding of the social and epidemiological context. Intervention units defined according to (i) shared sleeping spaces and (ii) household membership, showed substantial limitations as it was not possible to define them clearly and they were extremely variable within the study setting. Incorporating local definitions and community preference in the trial design led to the appropriate intervention unit—the compound—defined as an enclosed space containing one or several households belonging to the same extended patrilineal family. Our study demonstrates the appropriateness of using transdisciplinary research for investigating alternative intervention units that are better tailored to reactive treatment approaches.

## Introduction

Following a significant decline in malaria transmission, infections become increasingly clustered in “households or groups of households which maintain higher levels of transmission” than the rest of the population (1, 2). In these clusters, both incidence of clinical malaria and prevalence of infection would be higher than in the surrounding areas (3, 4). Targeting geographical clusters of both clinical and asymptomatic infections could therefore decrease the human reservoir of infection and thus further reduce malaria transmission (5–9).

In low-transmission settings, progress toward malaria elimination may be achieved by surveillance-based approaches, such as reactive interventions (3, 8). Similar to contact tracing for infectious diseases such as Ebola, tuberculosis, and coronavirus, reactive interventions are triggered when an “index clinical case” is diagnosed at a health facility, initiating a visit by health workers to the symptomatic case's household and/or surrounding households for the screening and/or (presumptive) treatment of family members and neighbors (7). Given the centrality of the “household/s” in the approaches, its conceptualization remains intricately tied to the unit of the intervention (10, 11). The household constitutes the central unit of analysis for most surveys [such as the health and demographic surveillance systems (HDSS)] and types of interventions but is seldom critically examined, reflecting the dominant approach in reactive interventions and more generally in demography (10, 12). Social diversity in the composition of the household (13, 14), i.e., how it is socio-culturally, politically and economically defined/constituted and the implications of such definition for its analysis) are often not considered *a priori*.

Reactive interventions such as reactive case detection (RACD) has been widely implemented across various epidemiological settings, including those that recently attained or are closer to malaria elimination (3, 8). A key issue influencing the efficiency of the RACD is the spatial range of the unit of intervention, frequently referred to as the “radius of the intervention.” Typically, for RACD, everybody living in the index clinical case household is screened and treated if positive or treated regardless of their infection status. However, the spatial range and the extent to which other households are considered for screening or treatment varies (3, 15–18). Determining this geographical parameter largely follows an epidemiological and operational perspective (3, 15, 19). Epidemiological factors are defined according to local transmission, vector species, environment which determine the geographical boundaries used to identify those at risk or vulnerable to infection (20–23). The operational factors pragmatically consider the ability of the local health systems to implement RACD in terms of availability of resources (funding, human resources) (3, 15, 18, 24). The latest Global Technical Strategy (2016–2030) for malaria, which reinforces calls for eradication by setting out the vision for a malaria free world, describes the spatial range for RACD approaches as *the most efficient, sensitive, and feasible radius for testing around the index malaria case*, depending on *the epidemiology and the local health system* (24). This perspective allows for the adaptation of the intervention unit according to the local setting in terms of disease transmission and health system factors but abstracts it from the social context. Protocols and research for case investigations in RACD vary widely, with often limited evidence for, or justification of, the decisions guiding the geographic scope of the intervention (3, 15, 19). Previous research has shown that social variability, i.e., migration, livelihoods, housing structure, residence, and sleeping patterns, within which they occur play a key part on the effectiveness of biomedical interventions (25–27). Nonetheless, to date, there are no studies that have explored how social context influence RACD approaches; community involvement in defining what is the appropriate targeted intervention unit has been rarely investigated. Given the diverse and complex pathways which affect malaria risk and disease transmission, addressing this gap remains crucial toward a better understanding of the appropriate intervention unit for RACD approaches.

### The Study Protocol and Related Assumptions

As researchers, we were involved in a novel approach where transdisciplinary research was used to assess and improve the effectiveness of RACD to target asymptomatic malaria-infected individuals (secondary cases), part of which consisted of establishing whether the household should be the unit of intervention as initially proposed in the study protocol. The transdisciplinary study conducted in The Gambia was centered around a cluster randomized trial (CRT), reactive household-based, self-administered treatment (RHOST) (ClinicalTrials.gov, NCT02878200, 25/08/2016) evaluating a new RACD approach that combined: (i) the passive detection of clinical malaria cases; (ii) systematic treatment with dihydroartemisinin-piperaquine (DHAP) of all household members sharing the same sleeping area with the index clinical case, without screening for infection; and (iii) an active community participation strategy involving patients, their households and other community actors as stakeholders in the intervention strategy (28). The trial was planned over two transmission seasons. In the first season (preparatory phase), 17 intervention and 17 control villages were identified and approaches to integrate the intervention into the communities and the health system were tested and adapted through formative research. During this process, trial implementation concerns were identified and solutions co-developed with relevant stakeholders, including community members, health service providers and policy makers. According to the original design, a clinical malaria case (index case) from an intervention village, after being diagnosed at the local health facilities or by village health workers, will be given a sufficient amount of DHAP to treat all members of their household. One key challenge of the approach was the definition of the intervention unit (i.e., treatment unit for epidemiological impact), which lies with the conceptualization of the household.

Maintaining the current dominant scientific paradigm of standardized households may shadow alternative contextualized solutions which addresses the realities of the concept of the household (29, 30). To date, household definitions remain influenced by the United Nations (UN) household guidelines for census enumerations based on the three main criteria of (i) residence (i.e., living together/sleeping under the same roof); (ii) housekeeping (i.e., pooling of resources), and (iii) provision of food (i.e., eating from the same pot). For post-colonial states like The Gambia, the adoption of the UN term of “household” and its corresponding guidelines for their national statistical systems became a measure of their transformation into “modern states” and of the attainment of their development goals as evaluated by the international community (31). The UN approach has been criticized for being Eurocentric as its premise is built around the organization of small nuclear families which often differs from the dynamic living arrangements in much of sub-Saharan Africa (10, 31).

To address fundamental tensions that arise between the standardized household concept as proposed by the UN and contextually variable local residence patterns and social organization, countries slightly modified the UN definition (31). For instance, the 2013 Gambian population and housing census defines the household “*as a person or group of persons who live together in the same house or compound, share the same house-keeping arrangements and are catered for as one. It might be worth noting that members of a household are not necessarily related by blood or marriage as the case of maids in some instances”* (32). The definition maintains focus on the UN criteria of housekeeping and provision of food, with slight “adaptation” of residence to the national context. Nonetheless, for comparability purposes, the national definition remains problematic since it also implies a standardized unit, which in practice, remains flexible. Comparisons for the purpose of research could be made with standardized surveys which in contrast to national censuses collects more in-depth data at the household level. For instance, the HDSS set up in the study area, collects data on demographic and population-based health indicators at the household level to support and inform clinical studies (33). The HDSS defines the household as “*a person or group of persons living in the same house or compound, sharing the same cooking arrangements”* (33). Although the definition also accounts for residency, it does not explicitly state the role of kinship relations in defining household membership.

Household variability include social rules governing marriage and family, kin and non-kin obligations and other political and economic aspects, and may manifest to a significant degree within the context of intra-household relations (10, 12, 34, 35). Its definition can rarely be standardized and for its appropriate application in research, it may be more useful to consider the social dynamics affecting specific aspects related to the goals of a specific intervention (34). Our study therefore aimed to identify the relevant social contextual factors for defining an appropriate intervention unit for the RACD approach.

## Methods

### Study Setting and Population

This transdisciplinary study was carried out during the preparatory phase of the trial in the North Bank region of The Gambia. Study villages were mainly populated by Fula, Mandinka, and Wolof ethno-linguistic groups, with a minority identifying themselves as Bambaras, Turkas, and Tilibonkas who migrated from neighboring Mali, Guinea, and Burkina Faso, respectively. The population is mainly Muslim. Most of the villagers were engaged in cash-crop and subsistence farming and, to a lesser extent, herding for livelihood. Peanuts are the main cash-crop while rice, maize, beans, and vegetables are grown as subsistence crops. Migration into the area is common during the rainy season when demand for labor in agricultural practices is high. The pursuit of socioeconomic advancements has contributed to young men migrating out of the area, mainly to Europe, influencing the local economic and social life. The economy of a significant number of local families are supplemented by remittances received from relatives abroad.

### Malaria Transmission

The Gambia is one of seven countries in West Africa that has achieved significant progress toward malaria elimination (36). The progress has been attributed to the scale-up of standard control measures, that include case management with artemisinin-based combination therapy (ACT) and Long-Lasting Insecticide Treated Bed nets (LLINs) (37). Nevertheless, malaria transmission remains spatially heterogenous, with two major strata, low transmission in western and central Gambia and moderate transmission in eastern Gambia. The study setting was considered a low transmission area, with malaria prevalence of <5% (36). *Plasmodium falciparum* is the main malaria species in the area.

### Study Design

As part of the formative research, a qualitative study based on ethnographic methods was carried out in continuous dialogue with field epidemiological investigations. The research strategy used an emergent theory design (38) wherein new insights from on-going data collection aimed to nurture existing theory in the two disciplines involved. The research team consisted of social scientists and fieldworkers. All social scientists had previous research experience in the region. The fieldworkers, also acting as translators, had received training and were experienced in social science research. The research was carried out in collaboration with a larger inter-disciplinary team of epidemiologists, health system, and health economics researchers.

### Data Collection

Fieldwork conducted between March–December 2016 facilitated data collection through in-depth interviews (*n* = 88), participant observation [including informal conversations (*n* = 9)], participatory group discussions (*n* = 10), cases studies (*n* = 10) and review of trial reports. Data from the different methods were triangulated to confirm, challenge and deepen the validity of the conclusions that either component might yield alone.

### Sampling

A purposive sample of study participants was included and access to the informants was facilitated through snowball sampling. The latter increased confidentiality and trust with respondents and further improved reliability in the collected data. Interviews were carried out in English and translated into the language of the respondent by trained fieldworkers. All responses were translated back in English to the researchers. Interviews were all recorded and transcribed verbatim by the translators. Additionally, fieldnotes were written during interviews by the researchers or immediately after the interviews.

### Data Analysis

An iterative process of analysis was performed concurrently with data collection. Investigators conducted preliminary analysis together whilst in the field and these findings were translated into question guides for follow-up interviews. Final transcripts and fieldnotes were systemized and analyzed thematically in NVivo 11 Qualitative Data Analysis software (QSR International Pty Ltd. Cardigan UK).

### Ethics

Approval, for the study, including oral consent, was obtained from the Gambia Government/MRC Joint Ethics Committee and by the Institutional Review Board of the Institute of Tropical Medicine, Antwerp, Belgium. The interviewers followed the Code of Ethics of the American Anthropological Association. All interviewees were informed before the interview about the topic and types of questions and their right to decline participation, to interrupt or withdraw from the research. Oral consent was sought before each interview and was documented by the interviewer. Oral consents were preferred as the risk to the participant was minimal and the act of signing one's name on a document could create mistrust since it is not customary practice within the local communities. Interviewees' confidentiality was assured by assigning unique identifiers to the collected forms.

## Results

We present how the RACD intervention unit was operationalized by outlining the different options considered during the trial implementation. The first step was a pilot of the intervention at the smallest treatment unit possible and then, adapting the strategy based on an emergent understanding of the social and epidemiological context.

### Operationalizing the RACD Intervention Unit

#### The Sleeping Space as Treatment Unit

Initially, the “sleeping space” was chosen as the smallest possible treatment unit, being potentially the most feasible. Treatment based on sleeping spaces was operationalized as targeting “people sleeping in the same room with a clinical index case.” Targeting the physical unit where people sleep was considered relevant based on (i) epidemiological evidence of malaria clustering around a clinical case (3, 4); (ii) the consideration that malaria treatment distribution was more feasible when limited to a smaller number of persons, and (iii) the implicit assumption that sleeping areas and who sleeps where was easily determined and stable. The following issues in identifying individuals eligible for reactive treatment based on targeted “sleeping areas” were the most common.

##### Unclear Unit Definition

Challenges with defining the spatial unit of the intervention were related to (i) difficulties in defining sleeping spaces within a room vs. the house; and (ii) entomological evidence on the influence of housing structures on malaria transmission (39–41). In the study area, the common housing structures were: (i) traditional single-room mud houses with thatched roofs and open eaves; and (ii) multi-room cement line houses with corrugated roofs. In the former, there was a single enclosed space which served several functions, including for sleeping. In the latter, multiple adjacent rooms often separated by walls that did not extend to the roof served as sleeping areas. These different housing structures co-existed in intervention villages, suggesting that our initial definition of a “sleeping area” was heterogeneous and not very practical for implementing the intervention.

##### Flexibility of Sleeping Patterns and Intra-Household Mobility

Sleeping patterns within rooms were very heterogenous and flexible. Sleeping patterns were largely based on social rules on kinship and marriage (42–44), wherein the simplest model was: “the husband sleeps with his male children in one room, while his wife sleeps with the mother-in law and female children in another room.” However, in a compound with several houses, each of them was occupied by an adult male and his family [wife/s and child(ren)]. The sleeping arrangements for children depended on their age and gender, with children under 5 years sharing a sleeping room with their mother. For older children, the females shared a room with their mothers while the males shared with their father or had a separate room (i.e., so-called boy's rooms). In most polygamous marriages (45), the husband would have an individual room where wives took turns to sleep. Although these arrangements present a systematic pattern, changes to the location and persons staying in these rooms were frequent.

These changes were mainly for social reasons such as when children moved from their mother's to grandmothers' room due to illness or to accommodate visitors (42). Same-gender sleeping arrangements were the norm for adults.

“*If I have stranger (guest) who I know, you know it can be my friend, then I sleep with them in my room, but if it is a child, he/she sleeps with the children. If the bed is small for them, they spread down to sleep while the stranger (guest) will sleep on the bed.”* (Household head, in-depth interview)

Based on these arrangements, children—mostly boys—were most likely to move across sleeping places in the house. Changes in sleeping locations were also affected by the season; household members slept outdoors due to hot weather. Such intra-household mobility and changes in sleeping locations occasionally affected the identification of participants eligible for treatment. For example, in an index-case household, some people who normally slept in the same room with the index case but temporarily moved to another room were missed for treatment based on the trial criteria of locating persons based on fixed sleeping spaces. Considering these challenges highlighted for treating “people sleeping in the same room with a clinical index case,” from the trial's perspective, the next logical step to consider as treatment unit was “all members of the household of the clinical index case.”

#### The Household as Treatment Unit

Targeting all household members living with the clinical index case assumed that (i) the household was clearly defined which was; (ii) ideal for scaling up the intervention as people could easily name all persons living in their household. Our ethnographic findings showed that the local household was largely understood within the context of social organization and centered on patrilineal kinship relations and virilocal patterns of residence (i.e., women leaving to reside in the village of the father of their husbands) (45–47). Gender roles provided men a predominant position within the family, defining them as household heads, and considered as the leaders of the therapy management group, i.e., individuals who took charge of therapy management with or on behalf of the sufferer (48–50). The household head and other senior family members could identify household members, including at least two generations of extended families (e.g., mother, brother, their wives and children). This group of people were described as normally living together and eating from the same cooking pot (i.e., common cooking arrangement) (10). The local definition, however, had a limited interpretation that moved away from the initial trial approach of capturing all persons living in the same household with a clinical index case. Its implication for the treatment unit became particularly relevant when considering that seasonal migration during the rainy (malaria transmission) season was a common activity in the area.

##### Seasonality and household composition

In most communities, rural households need additional labor for peanut farming (50). Therefore, the farming period accounted for short-term migration of seasonal workers from neighboring villages or other countries. Two categories of seasonal workers were observed which affected household compositions and further gave meaning to de-facto membership (actually living in or considered to be a permanent member of the household). The first category of workers were the members of the household who returned temporarily to support the family on the farms. These were mostly persons that had moved for economic reasons to urban areas within the country or elsewhere in Africa (45, 51). Although mostly absent during the year, migrant sons were regarded as members of the household due to the kinship relations, and their financial contribution to the household through remittances. During the time of their visit, the migrant sons, if unmarried would sleep in the father's room or boy's room, and if married slept in their own house within the compound.

The other category of short-term migrants were hired workers (45, 52) also referred to as *surga* or *mbedan*. Based on the availability of land and financial strength, household heads hired seasonal workers with different payment agreements, i.e., the *surga* received a piece of land they could cultivate while working in the owner's fields, and the *mbedan* worked for a pre-stablished amount of cash. These *surgas* and/or *mbendans* often shared the household accommodation either with the older males or in separate rooms.

“*You know this is the same compound if I catch a surga and my brother happens to catch another surga we look for a separate house for them to occupy in the compound*.” (Adult man, farmer, in-depth interview)

Our findings reveal that the presence of seasonal workers during the agricultural period was common in the study villages. Nevertheless, when asked to list the members of their household, the household head does not mention short-term migrants unless specifically enquired for (35). The findings resonate with previous literature, which show that households are largely made up of individuals related by blood or marriage (11, 13, 52). These cultural explanations give insights into migrants' integration within households and compounds, including implications for the enumeration of those targeted for treatment. In the context of declining malaria, when the majority of imported cases are often related to seasonal and long-term migrants (53), it is essential to question whether a targeted treatment unit based on residency criteria alone was an efficient approach.

#### The Compound as Treatment Unit

Beyond the household, the compound was relevant as a socio-spatial unit for the intervention. Epidemiologically, it was a larger spatial unit with clusters of people with similar exposure to mosquito bites and treatment-seeking behavior (28, 54). The compound also accounted for the social structures and local residence patterns mostly based on kinship and marriage relations (47, 55). Compounds within the village were typically characterized as an enclosed space of one or several households belonging to the same extended patrilineal family (47). Informants described several variations of the basic structure of social organization depending on its size (47). Nonetheless, the compound was clearly identified as the largest unit of residence, therapy and production unit in which authority was exercised (55) and accounted for all household members.

Operationally, treatment at the level of the compound seemed a much clearer target in terms of the potential for scaling up. Compounds addressed the issue of intra-household mobility in sleeping arrangements but also provided stability in allocations as people rarely slept out of their compounds. Nevertheless “treating all compound members of a clinical index case” was operationally complex due to the larger number of persons to be treated, on average 15.6 persons/per compound.

##### Malaria risk perception

Our key community group discussions and interviews revealed further limitations with restricting treatment to those sharing sleeping spaces or living in the same household with a clinical index case. Community responses toward defining the treatment unit were varied and related to their perception of malaria risk.

“*If my household takes the medicine, we are still not fully protected because we still mingle with other household members (eat together, share utensils), chat outside late at night with other villagers.”* (Household head, in-depth interview)

Informants expressed that people, particularly young men, from different households (from the same compound, from the same village and even from different villages) stayed outdoors until late at night (several participants specified “until 1 a.m.”). They pointed out that the risk of contracting and transmitting malaria went beyond their household, to include the compound and the village. In case of households, most informants that received treatment considered themselves and others (who they slept together with and took the medicine) within their household as protected from malaria. Informants explicitly mentioned that although all those who slept together with the malaria patient were protected, other untreated persons within the same household and compound, other surrounding compound and the village at large remained at risk of malaria.

“*Everyone in the compound should be treated, because if treatment is given to those who sleep together whilst they still stay with others that don't have treatment, it means they are still not fully protected.”* (Caretaker of index case, in-depth interview)

Some respondents insisted that it would be a good intervention to provide treatment at the compound or even at the village level, for general prevention for all. These expressions for mass treatment beyond the household and compound unit by the community members were often made in reference to previous mass drug administration programs for malaria implemented in the area (26, 56, 57).

## Discussion

Our research aimed to identify relevant social contextual factors for defining the appropriate intervention unit (i.e., treatment unit) for RACD—which departed from the commonly-used household as the key analytic unit. Our findings on the rural Gambian household reinforces the notion that there is no one-size-fits-all definition (10, 13, 34, 55), and highlights tensions between local realities and standardized ideals crucial to RACD and more generally epidemiological approaches. The findings confirm that for interventions focused on the household level, on which policy decisions are frequently based, relevant social dynamics such as living arrangements must be well-understood (31).

Within the study setting, variation in sleeping patterns and household membership reflecting local realties were commonplace, presenting a mismatch with universalist assumptions. As observed in other sub-Saharan African settings, flexibility in sleeping patterns is often necessary to accommodate guests whilst also contributing to the early childhood socialization (58–60). The observed variations in sleeping patterns contributed to the complexity of identifying people for the intervention based on where they slept. Further contributing toward this complexity were local understandings about who is a member of the household. The exclusion of non-kin, non-blood relations such as seasonal workers in the local household definition was explicit in our data. On the other hand, children who left home, but maintained financial contribution and collaborated in productive agricultural work remained included. This highlights the importance of the phrasing of questions surrounding the household and the compounds in surveys and raises questions about accuracy of demographic and socio-economic data given the high migration levels of Gambian men (61).

Given the fluidity surrounding the concept of the household, in practice, the compound was highlighted as a better-defined treatment unit, because it signified (i) a clear spatial residential unit which accommodated most household members, (ii) a productivity unit which fostered cooperation and solidarity amongst household members, and (iii) included a therapy management group to enable the distribution of treatment. Previous research shows the relevance of the compound for adequately capturing social dynamics, including residency patterns which remains crucial for health interventions (33, 47). The corresponding advocacy by some community members for mass biomedical treatment at village level should not be ignored. Such advocacy creates tension with the basic premise of this intervention as the RACD approach for the trial was conceptualized based on the call for more targeted approach for malaria elimination (9). However, their preference could be understood within the backdrop of community trust with biomedical interventions and the availability of vital therapeutic opportunities (62). Before the RACD intervention, a most recent MDA for malaria was implemented in the area by the Medical Research Council, the Gambia (MRCG) (26). Moreover, this research institute was perceived as an alternative health provider within an extremely limited health system. The relationship between the MRCG and communities in the Gambia and its implications for trust in research has been extensively discussed (26, 62–66).

Our findings highlight that even within an environment which fosters trust in biomedical interventions and wherein communities are engaged, tensions can still exist between biomedical rationale of implementing an intervention (i.e., designing a more targeted contextualized intervention for impact on malaria transmission) with community interests (i.e., in terms of addressing their perception of malaria risk to beyond defined boundaries). Addressing this issue, requires the continuous use of co-creative systemic research approaches (29), which acknowledges that community members and researchers both bring valuable insights during the research process, and that knowledge ought to be generated collaboratively (67). Community members have detailed knowledge on their contextual realities that shape their risk perceptions, whilst researchers contribute toward the research methods and methodologies. This complementary approach gives further insights on the avenues for building bridges between the primary aims of research with community concerns on its relevance.

Attempts to standardize and pre-define a complex, fluid and essentially subjective concept of the household has implications for the way household composition and residence are understood (68). Variability in household definitions have been accounted for during survey designs, wherein the tools are attuned to capture the diverse realities whilst still maintaining standardization for comparability purposes (10, 68). Moreover, it is acknowledged that if contextualized household definitions are clearly stated, including its implications, this could counter misunderstandings and therefore lead to its more informed analysis (31, 34, 55). Such a perspective can also help us understand why it is important to consider and address social variability in interventions such as RACD-type approaches. This RACD intervention considers the unique transmission patterns of our study setting, similar to some parts of sub-Saharan Africa were malaria clustering occurs mainly within local villages or compounds (1, 2).

## Conclusion

For this study, social variability at the household level was addressed through a transdisciplinary approach which considered and understood what works for the people and worked for the project. This was facilitated by asking the relevant questions and using emerging findings from ethnography and community participatory discussions to improve epidemiological outcomes and address epidemiological concerns for implementation as they emerge. Our findings show that, with this approach, RACD intervention units, can be appropriately tailored to local realities.

## Data Availability Statement

The raw data supporting the conclusions of this article will be made available by the authors, without undue reservation.

## Ethics Statement

The studies involving human participants were reviewed and approved by approval, for the study, including oral consent, was obtained from the Gambia Government/MRC Joint Ethics Committee and by the Institutional Review Board of the Institute of Tropical Medicine, Antwerp, Belgium. Written informed consent for participation was not required for this study in accordance with the national legislation and the institutional requirements.

## Author Contributions

FJ, JR, and KP conceptualized the study. FJ and JR designed the experiments and mainly collected and analyzed the data. FJ wrote the manuscript and KP contributed as well. YM, JO, UD'A, JA, JB, RG, JR, and KP edited and reviewed the manuscript. All main authors read and approved the final manuscript.

## Conflict of Interest

The authors declare that the research was conducted in the absence of any commercial or financial relationships that could be construed as a potential conflict of interest.
